# Nitrofurans: Revival of an “old” drug class in the fight against antibiotic resistance

**DOI:** 10.1371/journal.ppat.1009663

**Published:** 2021-07-08

**Authors:** Vuong Van Hung Le, Jasna Rakonjac

**Affiliations:** 1 School of Fundamental Sciences, Massey University, Palmerston North, New Zealand; 2 Maurice Wilkins Centre, University of Auckland, Auckland, New Zealand; Tufts Univ School of Medicine, UNITED STATES

## Introduction

Antibiotic resistance is one of the greatest contemporary threats to the human health, which has increasingly been undermining the effectiveness of existing antimicrobial therapies. Development of novel antibiotics is inarguably the key to combat this threat, yet this is a lengthy process (10 to 15 years) at a hefty price tag of approximately US$1.3 billion for development of an approved drug [1,2]. A new drug, once introduced into the market, also faces the risk of drug resistance emergence. It is, therefore, essential to diversify therapeutic strategies, including revival and reintroduction of “old” antibacterials for treating multidrug-resistant pathogens [3]. Nitrofuran class of synthetic molecules, introduced in the 1940s and 1950s, belongs to this category [4]. Several nitrofurans are currently on the market: nitrofurazone for topical infections and urinary catheter coating, nitrofurantoin for urinary tract infections, and furazolidone for bacterial diarrhea and *Helicobacter pylori* infections. Here, we highlight aspects of this drug class that have recently been unraveled, laying foundation for future improvements and judicial uses of nitrofurans against ever-expanding antibiotic-resistant bacteria.

### Nitrofuran-activating enzymes in *Escherichia coli*

**Fig 1:**
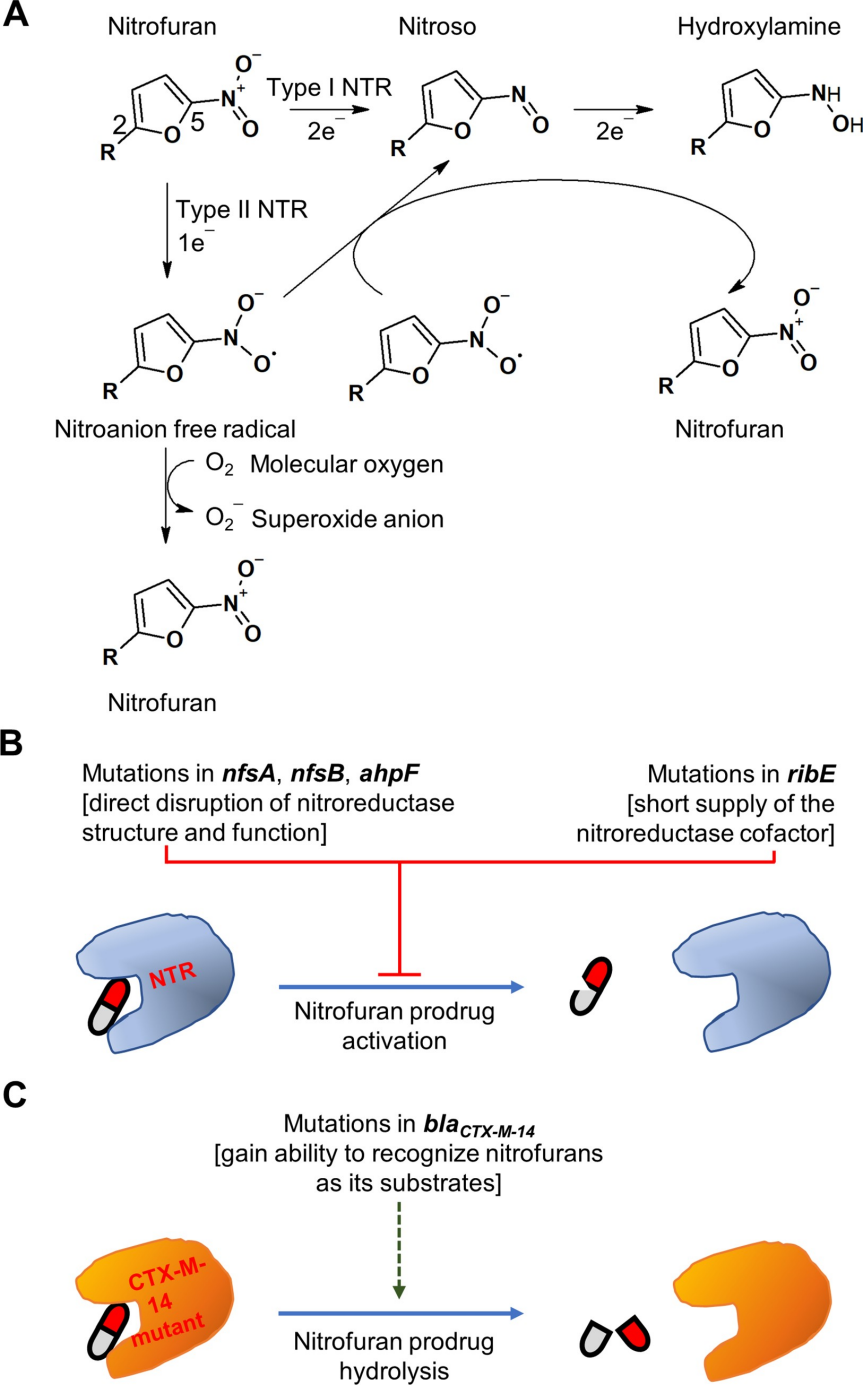
Mechanism of nitrofuran activation and resistance. **(A)** Schematic pathway of nitrofuran reduction by NTR enzymes. C_5_ and C_2_ of the furan ring are numbered. **(B)** Nitrofuran resistance by mutations that disrupt prodrug activation. **(C)** Nitrofuran resistance mechanism by a mutant of β-lactamase CTX-M-14 (*bla*_*CTX-M-14*_) that catalyzes nitrofuran hydrolysis. NTR, nitroreductase.

Nitrofuran compounds are prodrugs. In *E*. *coli*, they are activated via reduction by 2 type I oxygen-insensitive nitroreductases, NfsA and NfsB. These enzymes catalyze a stepwise 2-electron reduction of the nitro moiety into reactive nitroso and hydroxylamino derivatives, one of which is considered responsible for the antibacterial activity of nitrofurans (Fig 1A) [5–7]. Peterson and colleagues reported the existence of type II nitroreductase activity in *E*. *coli* that reduces nitrofuran by 1-electron transfer mechanism and is sensitive to molecular oxygen (Fig 1A) [8]. The identity of enzymes catalyzing the oxygen-sensitive nitrofuran reduction has not been revealed until 2019, when we identified a novel nitrofuran-activating enzyme by selecting for furazolidone-resistant mutants in an *nfsA nfsB E*. *coli* double knock-out strain. A total of 15 independently isolated mutants resistant to bactericidal concentration of furazolidone contained mutations in the *ahpF* gene, which encodes a component of the antioxidant alkyl hydroperoxide reductase [9]. Subsequent enzymatic assays of purified AhpF protein determined that this enzyme is a type II oxygen-sensitive nitroreductase [9].

The antibacterial mechanism of the nitrofuran derivatives, once activated by nitroreductases, is ill defined. Multiple effects have been observed, including DNA lesions and oxidative stress and inhibition of the RNA and protein biosynthesis [10–12]. However, it remains to be clarified which of these affected targets (DNA, RNA, and protein) are directly attacked by the nitroreductase-activated nitrofuran derivatives and which are simply downstream effects of the interaction between these derivatives and bacterial essential targets. We hypothesize that the nitro moiety at the C_5_ of the furan ring, once reduced by nitroreductases, acts as a warhead to covalently modify the targets, while the side chain at the C_2_ of the furan ring defines its selectivity, depending on how well it fits into the ligand-binding pocket of a target. Future experiments looking into the interaction between nitrofurans and individual *E*. *coli* essential proteins (approximately 300 in total) in the presence and absence of a nitroreductase may shed light on the nitrofuran cognate targets.

Existence of potential unknown nitrofuran-activating enzymes in *E*. *coli* presents another interesting question for this antibiotic class. The observations that nitrofurans still retain antibacterial activity against the triple *nfsA nfsB ahpF* mutant, either individually or in a synergistic manner with sodium deoxycholate, an antimicrobial bile salt [9,13], point to the existence of these enzymes, although contribution of these enzymes to the overall antibacterial activity of the nitrofurans (nitrofurantoin and furazolidone) assayed in these studies may not be remarkable. The modest effect on nitrofuran minimum inhibitory concentration (MIC) of these unknown enzymes could be due to low protein expression or low affinity for the nitrofurans tested. Identification of these nitrofuran-activating enzymes in *E*. *coli* and understanding their biology (physiological functions, regulation of protein expression/action, and enzyme–nitrofuran molecular interaction) would provide useful knowledge to facilitate development of new nitrofuran-based antibacterial therapies. For example, in the case of potential low expression of the activating enzyme, structure-based virtual screens with an ultra-large library [14] targeting the expression regulator of the candidate enzyme or targeting the enzyme allosteric site can be employed to seek the adjuvant molecules that up-regulate the enzyme expression or enhance the enzyme activity, respectively; these adjuvant molecules can be used with nitrofuran antibiotics for improved antibacterial potency. Alternatively, rational design of nitrofuran analogs such that they favorably bind to the redox active site of the activation enzyme through *in silico* enzyme–drug docking simulation, followed by experimental validation, may generate novel nitrofuran candidates that are different from clinically used nitrofuran drugs in the terms of prodrug activation and resistance mechanisms. A similar strategy can be used to identify novel nitrofurans that are effective against bacteria outside of the spectrum of action of current nitrofurans, e.g., *Pseudomonas aeruginosa*.

### Nitrofuran resistance mechanisms in *E*. *coli*

Mutations of *nfsA* and *nfsB* are the major bacterial mechanism for gaining resistance to nitrofurans, in both laboratory and clinical strains of *E*. *coli*. There is one exception in which a de novo–selected nitrofurantoin-resistant *E*. *coli* strain had the wild-type *nfsA* and *nfsB* alleles but contained an in-frame deletion in the *ribE* gene that encodes an enzyme in the biosynthesis of flavin mononucleotide, an essential NfsA/NfsB cofactor (Fig 1B) [15]. Perhaps due to the reduced bacterial growth rate caused by the *ribE* mutation, mutations in this gene have not been reported in *E*. *coli* clinical isolates and are unlikely to pose a significant threat to the efficacy of nitrofuran treatment.

The inability to prepare for the unknown often leads to disastrous consequences. In this spirit, a possible novel nitrofuran resistance mechanism(s), besides well-known *nfsA*/*nfsB* mutations, must receive due attention and resources in order to sustain the utility of this drug class. While the prevalence of nitrofuran resistance among *E*. *coli* clinical isolates in recent epidemiology surveys around the world is still low [9], nitrofuran-hyperresistant isolates with the MIC higher than 128 μg/mL have been encountered [15,16]. This high level of resistance cannot solely be explained by mutations in the *nfsA*, *nfsB*, and *ahpF* genes and points to unknown resistance determinants that are already circulating in pathogenic strains.

**Fig 2:**
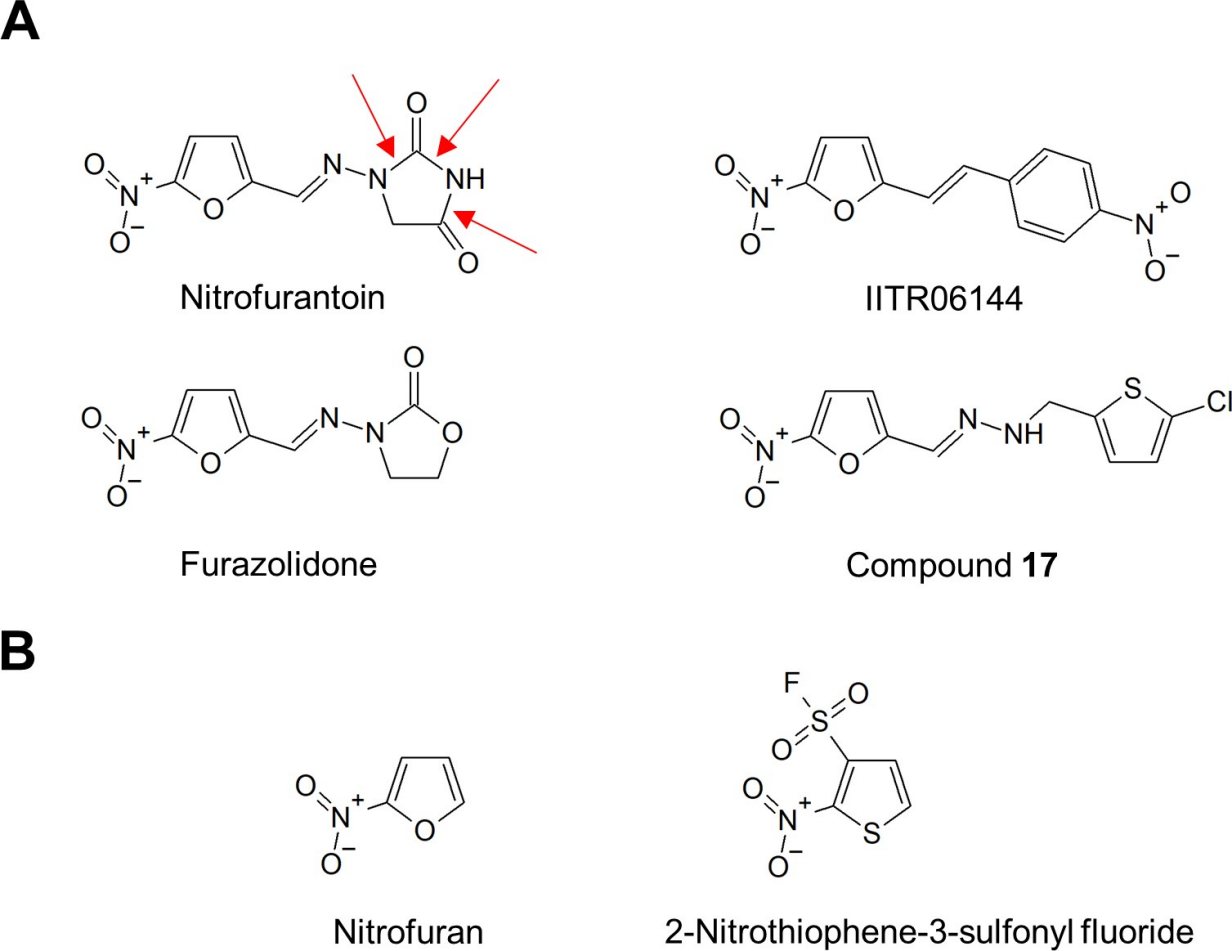
New nitrofuran molecules IITR06144 and compound **17 (A)** and a nitroaromatic pharmacophore 2-nitrothiophene-3-sulfonyl fluoride **(B)**. Red arrows indicate the amide bonds of nitrofurantoin that are hypothetically hydrolyzed by the CTX-M-14 triple mutant variant.

Of great concern is the reported nitrofurantoin resistance in *E*. *coli* uropathogenic clinical isolates in one hospital in North Wales (United Kingdom) in 2020 [17]. These isolates were found to have a mutated version of the extended spectrum β-lactamase CTX-M-14, which differs from the wild-type protein by 3 nonsynonymous changes (T55A, A273P, and R277C). When recombinantly overexpressed in an *E*. *coli* laboratory strain, the mutated CTX-M-14 enzyme caused hyperresistance to nitrofurantoin while retaining the ability to render β-lactam resistance [17]. The purified enzyme was able to hydrolyze nitrofurantoin in the *in vitro* enzymatic assay (Fig 1C). Although the exact hydrolytic products remain to be determined, we speculate that the triple CTX-M-14 mutant, by analogy to β-lactam drugs, may cut at the amide bonds in the hydantoin ring (Fig 2A, red arrows) such that the hydrolytic products have lower affinity to the activation enzymes or to the essential targets of nitrofurantoin. While the work is preliminary, this finding is very worrisome. The recent switch from trimethoprim/sulfamethoxazole combination to nitrofurantoin as the first-line therapy for urinary tract infections has majorly increased the exposure of *E*. *coli* to nitrofurantoin and will very likely select for this triple mutant of CTX-M-14. Threat of the CTX-M-14–mediated nitrofuran-resistant mutant is high, given that this is one of the 2 most predominant types of the extended spectrum β-lactamases globally [18]. Further investigation is urgently needed to understand the prevalence and expression of this CTX-M-14 variant that causes resistance to nitrofurantoin, the mechanism of nitrofurantoin hydrolysis by this variant and probably other β-lactamases, the potential of known β-lactamase inhibitors to block the hydrolyzing activity against nitrofurans. Additionally, *in vitro* evolution experiments with clinically important β-lactamase enzymes, directed toward nitrofurantoin hydrolysis and resistance, would predict what mutations may be selected under nitrofurantoin exposure in clinical settings. These efforts will help maintain the effectiveness of nitrofurantoin against the β-lactamase–positive *E*. *coli*.

### New nitrofurans for the future of infectious disease therapies

There have been very few attempts to develop next-generation nitrofuran antibacterial drugs in several decades. Over the past few years, however, work on nitrofurans has been revived, in parallel with their resurgence as an effective treatment option in the context of widespread resistance to other antibiotics. One prominent trend is to search for nitrofuran analogs that have a broadened spectrum and increased potency in comparison to the existing clinically used nitrofurans, such as IITR06114, a novel nitrofuran recently discovered in a small molecule screen [19], and compound 17 identified in a hit-to-lead optimization effort (Fig 2A) [20]. Extensive medicinal chemistry efforts have been undertaken to design novel antimycobacterial agents from the nitrofuran scaffold, culminating in a number of candidates with the submicromolar to nanomolar *in vitro* MICs [21,22]. It remains to be seen whether these nitrofuran candidates will be successfully brought to clinical trials in the years to come. Development of a new nitroaromatic pharmacophore, 2-nitrothiophene-3-sulfonyl fluoride, is also worth noting (Fig 2B). This new molecule has a similar molecular structure to nitrofurans and shares the mechanism of nitroreductase-mediated activation, providing a promising starting point from which novel potent drugs can be developed, including those that may be effective against formidable Gram-negative pathogens, such as *Acinetobacter baumannii* and *P*. *aeruginosa*, which naturally have high nitrofuran MICs [23].

Given that development of new drugs takes years, it is also important to conserve and enhance the effectiveness of already available nitrofurans. This can be achieved by combinations with other antimicrobial agents, capitalizing on interactions between them, either via synergistic interactions or collateral sensitivity. A synergistic interaction means that the antibacterial potency of 2 antibiotics, when used in combination, is stronger than the combined effect of the individual antibiotic when used alone. Such an effect was, for example, reported in the 2-way combinations of nitrofurans with the secondary bile salt deoxycholate [13], and vancomycin [24], and further enhanced in the triple combination of nitrofurans, deoxycholate, and vancomycin [25]. Meanwhile, another beneficial interaction that can be exploited is collateral sensitivity, where a resistance mechanism to one antibiotic confers increased sensitivity to another. For example, loss-of-function mutation of the protease-encoding *lon* gene causing tigecycline resistance results in hypersensitivity to nitrofurantoin [26]. Collateral sensitivity leads to a strategy of switching treatment from tigecycline to nitrofurantoin in sequential therapies to eliminate the strains resistant to the former, while increasing the chance of a successful bacterial clearance with the latter [26].

In contrast to high potency against *E*. *coli*, nitrofurans have very limited inhibitory effect, if any, on growth of *P*. *aeruginosa*, an opportunistic Gram-negative pathogen that causes respiratory system infections and urinary tract and soft tissue infections. In a screen for quorum sensing inhibitors from a library of Food and Drug Administration (FDA)-approved drugs, nitrofurazone was found to inhibit PqsE, a regulator of *P*. *aeruginosa* quorum sensing system. Nitrofurazone suppressed pathways regulated by this protein, including biofilm formation and production of virulence factor pyocyanin [27]. This finding opens an avenue for the repurposing of nitrofurans as antivirulence drugs to attenuate the severity of *P*. *aeruginosa* infections [28].

## Conclusions

The spread of antimicrobial resistance has led to a resurgence of nitrofurans, an old class of antibiotics. Recent studies have shed light on the mechanism of nitrofuran activation and raised urgent questions about emerging resistance mechanisms. Current efforts, such as search for improved nitrofuran derivatives, development of new pharmacophores, drug combinations, and repurposed uses of nitrofurans, hold a promise to make this drug class an important weapon in the combat against multidrug-resistant bacterial pathogens in the future.

## References

[1] Wouters OJ, McKee M, Luyten J. Estimated Research and Development Investment Needed to Bring a New Medicine to Market, 2009–2018. JAMA. 2020;323(9):844–53. doi: 10.1001/jama.2020.1166 32125404 PMC7054832

[2] O’Neill J. Securing new drugs for future generations: The pipeline of antibiotics. The Review on Antimicrobial Resistance. 2015. Available from: https://amr-review.org/Publications.html.

[3] Theuretzbacher U, Van Bambeke F, Canton R, Giske CG, Mouton JW, Nation RL, et al. Reviving old antibiotics. J Antimicrob Chemother. 2015;70(8):2177–81. doi: 10.1093/jac/dkv157 26063727

[4] Chamberlain RE. Chemotherapeutic properties of prominent nitrofurans. J Antimicrob Chemother. 1976;2(4):325–36. doi: 10.1093/jac/2.4.325 802123

[5] Zenno S, Koike H, Kumar AN, Jayaraman R, Tanokura M, Saigo K. Biochemical characterization of NfsA, the *Escherichia coli* major nitroreductase exhibiting a high amino acid sequence homology to Frp, a *Vibrio harveyi* flavin oxidoreductase. J Bacteriol. 1996;178(15):4508–14. doi: 10.1128/jb.178.15.4508-4514.1996 8755878 PMC178217

[6] Zenno S, Koike H, Tanokura M, Saigo K. Gene cloning, purification, and characterization of NfsB, a minor oxygen-insensitive nitroreductase from *Escherichia coli*, similar in biochemical properties to FRase I, the major flavin reductase in *Vibrio fischeri*. J Biochem. 1996;120(4):736–44. doi: 10.1093/oxfordjournals.jbchem.a021473 8947835

[7] Race PR, Lovering AL, Green RM, Ossor A, White SA, Searle PF, et al. Structural and mechanistic studies of *Escherichia coli* nitroreductase with the antibiotic nitrofurazone. Reversed binding orientations in different redox states of the enzyme. J Biol Chem. 2005;280(14):13256–64. doi: 10.1074/jbc.M409652200 15684426

[8] Peterson FJ, Mason RP, Hovsepian J, Holtzman JL. Oxygen-sensitive and -insensitive nitroreduction by *Escherichia coli* and rat hepatic microsomes. J Biol Chem. 1979;254(10):4009–14. 374406

[9] Le VVH, Davies IG, Moon CD, Wheeler D, Biggs PJ, Rakonjac J. Novel 5-nitrofuran-activating reductase in *Escherichia coli*. Antimicrob Agents Chemother. 2019;63(11):e00868–19. doi: 10.1128/AAC.00868-19 31481448 PMC6811407

[10] McCalla DR. Nitrofurans. In: Hahn FE, editor. Mechanism of action of antibacterial agents; Antibiotics. 5 / 1. Germany: Springer Berlin Heidelberg; 1979. p. 176–213.

[11] Bertenyi KK, Lambert IB. The mutational specificity of furazolidone in the *lacI* gene of *Escherichia coli*. Mutat Res. 1996;357(1–2):199–208. doi: 10.1016/0027-5107(96)00102-9 8876695

[12] Ona KR, Courcelle CT, Courcelle J. Nucleotide excision repair is a predominant mechanism for processing nitrofurazone-induced DNA damage in *Escherichia coli*. J Bacteriol. 2009;191(15):4959–65. doi: 10.1128/JB.00495-09 19465649 PMC2715711

[13] Le VVH, Olivera C, Spagnuolo J, Davies IG, Rakonjac J. *In vitro* synergy between sodium deoxycholate and furazolidone against enterobacteria. BMC Microbiol. 2020;20(1):5. doi: 10.1186/s12866-019-1668-3 31906851 PMC6945529

[14] Lyu J, Wang S, Balius TE, Singh I, Levit A, Moroz YS, et al. Ultra-large library docking for discovering new chemotypes. Nature. 2019;566(7743):224–9. doi: 10.1038/s41586-019-0917-9 30728502 PMC6383769

[15] Vervoort J, Xavier BB, Stewardson A, Coenen S, Godycki-Cwirko M, Adriaenssens N, et al. An *in vitro* deletion in *ribE* encoding lumazine synthase contributes to nitrofurantoin resistance in *Escherichia coli*. Antimicrob Agents Chemother. 2014;58(12):7225–33. doi: 10.1128/AAC.03952-14 25246406 PMC4249564

[16] Olivera C, Rakonjac J. Complete Genome Assembly of a Multidrug-Resistant New Delhi Metallo-beta-Lactamase 1 (NDM-1)-Producing *Escherichia coli* Human Isolate from a New Zealand Hospital. Microbiol Resour Announc. 2020;9(34). doi: 10.1128/MRA.00780-20 32816984 PMC7441242

[17] Edowik Y, Caspari T, Williams HM. The Amino Acid Changes T55A, A273P and R277C in the Beta-Lactamase CTX-M-14 Render *E*. *coli* Resistant to the Antibiotic Nitrofurantoin, a First-Line Treatment of Urinary Tract Infections. Microorganisms. 2020;8(12). 10.3390/microorganisms8121983 PMC7763680 33322113

[18] Bevan ER, Jones AM, Hawkey PM. Global epidemiology of CTX-M beta-lactamases: temporal and geographical shifts in genotype. J Antimicrob Chemother. 2017;72(8):2145–55. doi: 10.1093/jac/dkx146 28541467

[19] Bhando T, Bhattacharyya T, Gaurav A, Akhter J, Saini M, Gupta VK, et al. Antibacterial properties and in vivo efficacy of a novel nitrofuran, IITR06144, against MDR pathogens. J Antimicrob Chemother. 2020;75(2):418–28. doi: 10.1093/jac/dkz428 31665357

[20] Stevens M, Howe C, Ray AM, Washburn A, Chitre S, Sivinski J, et al. Analogs of nitrofuran antibiotics are potent GroEL/ES inhibitor pro-drugs. Bioorg Med Chem. 2020;28(22):115710. doi: 10.1016/j.bmc.2020.115710 33007545 PMC7914298

[21] Elsaman T, Mohamed MS, Mohamed MA. Current development of 5-nitrofuran-2-yl derivatives as antitubercular agents. Bioorg Chem. 2019;88:102969. doi: 10.1016/j.bioorg.2019.102969 31077910

[22] Agre N, Tawari N, Maitra A, Gupta A, Munshi T, Degani M, et al. 3-(5-Nitrofuran-2-yl)prop-2-en-1-one Derivatives, with Potent Antituberculosis Activity, Inhibit A Novel Therapeutic Target, Arylamine N-acetyltransferase, in Mycobacteria. Antibiotics. 2020;9(7). doi: 10.3390/antibiotics9070368 32630175 PMC7400135

[23] Sadlowski C, Park B, Araujo C, Das S, Kerr DL, He M, et al. Nitro Sulfonyl Fluorides are a new pharmacophore for the development of antibiotics. Mol Syst Des Eng. 2018;3(4):599–603. doi: 10.1039/C8ME00011E 30740245 PMC6366622

[24] Zhou A, Kang TM, Yuan J, Beppler C, Nguyen C, Mao Z, et al. Synergistic interactions of vancomycin with different antibiotics against *Escherichia coli*: trimethoprim and nitrofurantoin display strong synergies with vancomycin against wild-type *E*. *coli*. Antimicrob Agents Chemother. 2015;59(1):276–81. doi: 10.1128/AAC.03502-14 25348521 PMC4291362

[25] Olivera C, VVH L, Davenport C, Rakonjac J. In vitro synergy of 5-nitrofurans, vancomycin and sodium deoxycholate against Gram-negative pathogens. J Med Microbiol. 2021. doi: 10.1099/jmm.0.001304 33448923 PMC8346734

[26] Roemhild R, Linkevicius M, Andersson DI. Molecular mechanisms of collateral sensitivity to the antibiotic nitrofurantoin. PLoS Biol. 2020;18(1):e3000612. doi: 10.1371/journal.pbio.3000612 31986134 PMC7004380

[27] Baldelli V, D’Angelo F, Pavoncello V, Fiscarelli EV, Visca P, Rampioni G, et al. Identification of FDA-approved antivirulence drugs targeting the *Pseudomonas aeruginosa* quorum sensing effector protein PqsE. Virulence. 2020;11(1):652–68. doi: 10.1080/21505594.2020.1770508 32423284 PMC7549961

[28] Rampioni G, Visca P, Leoni L, Imperi F. Drug repurposing for antivirulence therapy against opportunistic bacterial pathogens. Emerg Top Life Sci. 2017;1(1):13–22. doi: 10.1042/ETLS20160018 33525812

